# Effective vemurafenib monotherapy for refractory Langerhans cell histiocytosis with sustained results post-withdrawal for over two years: a case report

**DOI:** 10.3389/fonc.2025.1688802

**Published:** 2025-10-09

**Authors:** Jiaxin Ren, Ling Gu, Xue Tang

**Affiliations:** ^1^ Department of VIP Clinic Nursing, West China Second University Hospital, Sichuan University, Chengdu, China; ^2^ Key Laboratory of Birth Defects and Related Diseases of Women and Children, Sichuan University, Ministry of Education, Chengdu, China; ^3^ Department of Pediatrics, West China Second University Hospital, Sichuan University, Chengdu, China

**Keywords:** Langerhans cell histiocytosis, mitogen-activated protein kinase, BRAF, vemurafenib, chemotherapy

## Abstract

**Background:**

Langerhans cell histiocytosis (LCH) is a rare, inflammatory myeloid neoplasm. Mitogen-activated protein kinase (MAPK) inhibitors, such as vemurafenib, can quickly control active disease in patients resistant to vinblastine and prednisone, but recurrence often occurs within a year after stopping treatment.

**Case presentation:**

We report the case of a 15-month-old girl with high-risk multisystem LCH and *BRAF^V600E^* mutation. The patient initially received treatment according to the LCH-III chemotherapy protocol but exhibited disease progression after two months of maintenance chemotherapy. Following initiation of vemurafenib monotherapy, the patient’s condition improved rapidly. The duration of vemurafenib monotherapy was one year and nine months. The patient remained disease-free for over two years after vemurafenib withdrawal.

**Conclusion:**

This case highlights the potential of MAPK inhibitor monotherapy for pediatric refractory LCH.

## 1 Introduction

Langerhans cell histiocytosis (LCH) is a rare and heterogeneous myeloid neoplasm that predominantly affects children, with an estimated incidence of five cases per million individuals (1). Although treatment with prednisone and vinblastine has significantly improved survival rates in patients with LCH, those classified as high-risk have a reactivation rate of 30% (2). Longitudinal studies have indicated that approximately 50% of patients exhibit resistance to prednisone and vinblastine, which results in disease progression and recurrence (3). Management of multisystem LCH (MS-LCH) refractory to vinblastine and steroid regimens remains a significant challenge. While nucleoside analogs, such as cytarabine, cladribine, or clofarabine, may present a potential therapeutic option for refractory LCH, these treatments are associated with increased chemotherapy-related toxicity (3–5).

Badalian-Very et al. documented that over 50% of patients with LCH have an oncogenic *BRAF^V600E^* mutation (6). Subsequent studies have further elucidated that beyond the *BRAF^V600E^* mutation, additional activating mutations within the MAPK signaling pathway are also present in LCH (7, 8). Therefore, MAPK inhibitors have been used for therapeutic management of LCH. Nonetheless, these inhibitors do not fully eradicate malignant clones, frequently leading to disease reactivation upon therapy cessation (9–11). In this report, we describe a case of LCH with a *BRAF^V600E^* mutation that progressed following prednisone and vincristine treatment. Notably, the patient achieved sustained complete remission for two years after the discontinuation of vemurafenib monotherapy.

## 2 Case description

**Figure 1:**
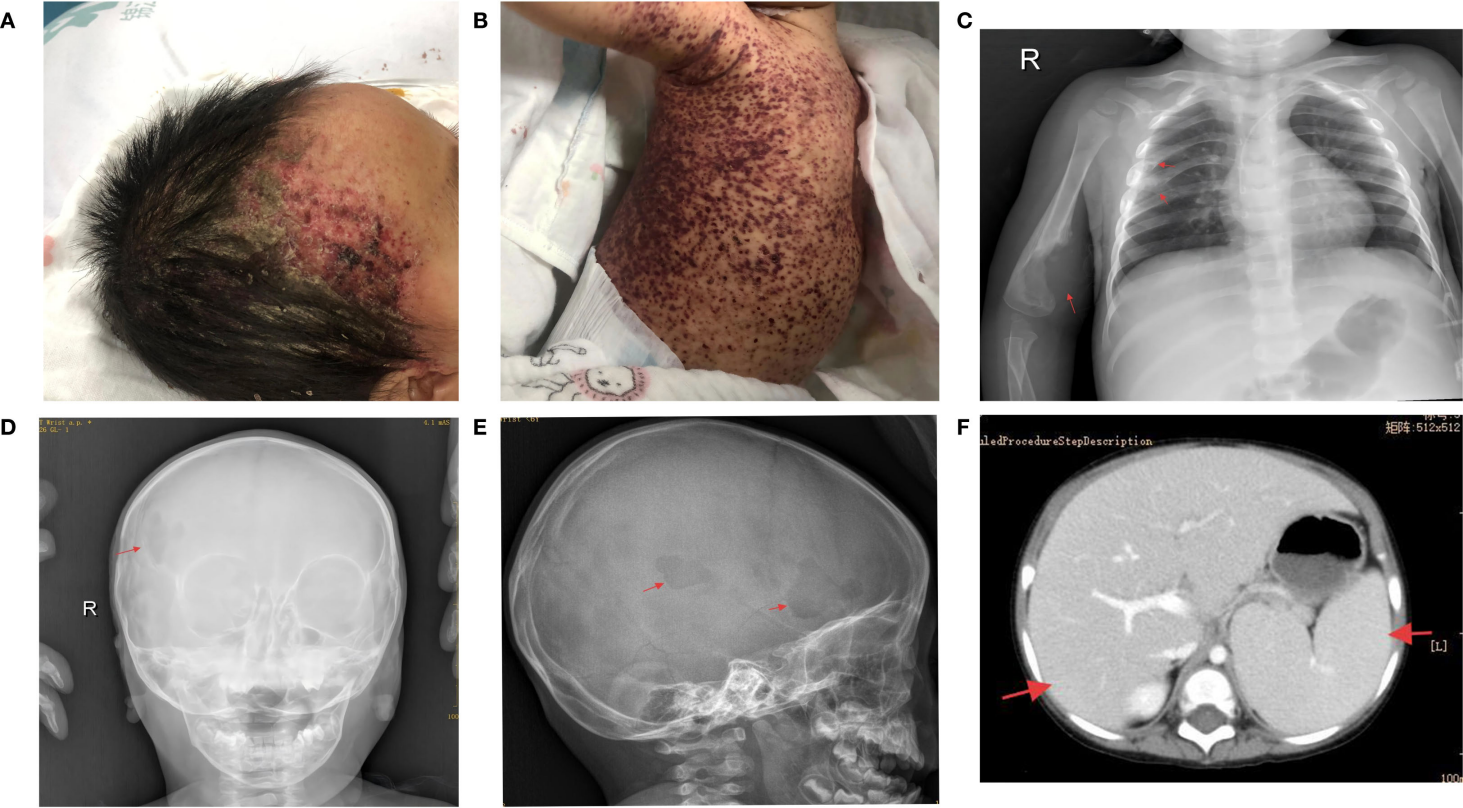
**(A, B)** Upon hospital admission, the patient exhibited purplish-red hemorrhagic rash and seborrheic rash on most of the trunk and head. **(C)** Chest X-ray showed bone destruction in the 4th and 5th ribs and right humerus. **(D, E)** Skull X-ray indicated bone destruction in the right frontal bone. **(F)** Abdominal enhanced CT revealed enlarged liver and spleen with reduced liver density.

A 15-month-old girl presented with a recurrent rash that persisted for more than six months. Initially manifesting near the hairline on both sides of the forehead, the rash progressively spread to the abdomen without any accompanying pruritus or exudation. She was diagnosed with purpura at a local hospital, and her condition improved following dexamethasone infusion. One month prior to admission to our hospital, the patient developed a painful, hard lump approximately 1 × 1cm in size on the right upper arm, characterized by an absence of fluctuation and normal skin temperature. Concurrently, the patient exhibited bilateral yellow-white otorrhea, along with a newly developed purplish-red hemorrhagic rash predominantly affecting the trunk, head, and perineum, with some bleeding and exudation on the right side (**Figures 1A, B**).

The patient was referred to the West China Hospital of Sichuan University for a skin biopsy to confirm the diagnosis. The results revealed atypical cells in the lesions that were positive for CD1a, S-100, and Langerin. In addition, a *BRAF^V600E^* mutation was detected in this lesion by polymerase chain reaction (PCR). The patient visited our hospital for further treatment. Physical examination revealed hepatomegaly (7cm below the costal margin and 8cm below the xiphoid process), splenomegaly (4cm below the costal margin), pallor, and a widespread rash. Routine blood analyses indicated severe anemia, with the lowest hemoglobin level recorded at 55 g/L, and thrombocytopenia, with the lowest platelet count at 22 × 10^9^/L. Liver function tests showed albumin, direct bilirubin, and γ-glutamyl transferase levels of 21 g/L, 17.8 µmol/L, and 91 U/L, respectively. Abdominal computed tomography revealed increased liver and spleen volumes and decreased liver density (**Figure 1F**). Chest and head radiographs revealed bone destruction in the 4th and 5th ribs, right humerus, and skull (**Figures 1C–E**). No lesions were found on the lungs by high-resolution chest CT. The *BRAF^V600E^* mutation was detected in both bone marrow and circulating cell-free (ccf) DNA with mutation abundances of 2.95% and 0.39%, respectively.

## 3 Diagnostic assessment, therapeutic intervention, and follow-up

**Figure 2:**
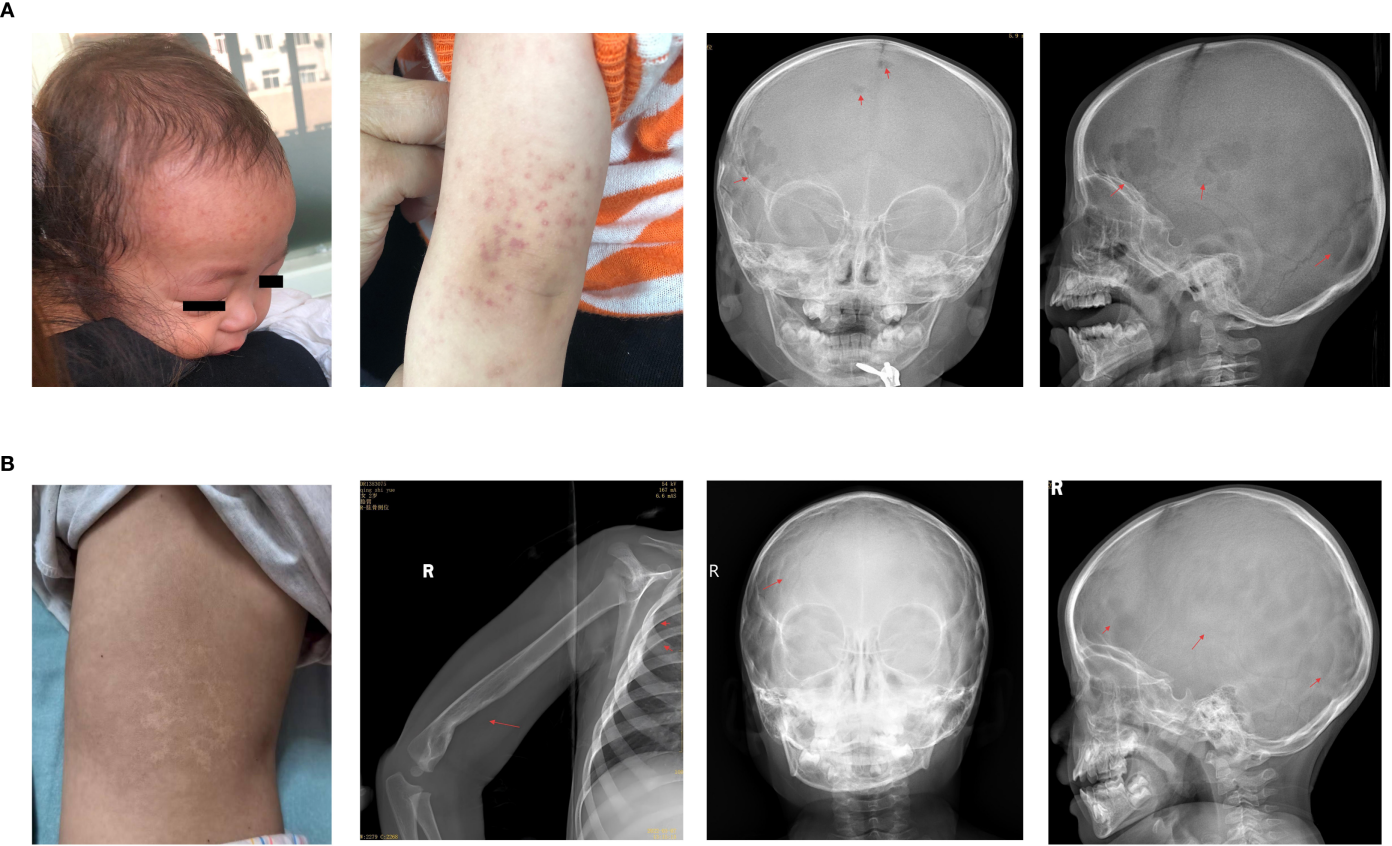
**(A)** During maintenance treatment, new rashes appeared on the forehead and arms, with enlarged lesions on frontal bone and new lesions on parietal bone. **(B)** The child showed slight back pigmentation, and the head, humerus, and ribs were mostly healed with no new lesions.

Based on pathological biopsy, the patient was diagnosed with high-risk organ involvement (RO+) MS-LCH. LCH III-directed induction chemotherapy was initiated, consisting of prednisone (40 mg/m^2^/day orally for days 1-28, with subsequent weekly tapering) and vincristine (2 mg/m^2^/day, intravenous bolus, on days 1, 8, 15, 22, 29, and 36). Vincristine was used as a substitute for vinblastine because of its unavailability in China. After 6 and 12 weeks of induction therapy, there was an improvement in anemia and thrombocytopenia, normalization of albumin levels, decrease in C-reactive protein (CRP) to normal levels, improvement in hepatosplenomegaly, and a reduction in bone lesions. The disease was initially assessed as active disease better following 12 weeks of induction chemotherapy and maintenance chemotherapy commenced on June 2, 2021. The mutational burden of *BRAF^V600E^* at the sixth and twelfth weeks of chemotherapy follow-up was 0.465% and negative (<0.05%), respectively. However, after two months of maintenance therapy, the patient’s condition was re-evaluated and was found to have progressed, as evidenced by a newly developed rash, enlargement of the original skull lesion, emergence of new bone lesions (**Figure 2A**), sustained increase in CRP levels (peaking at 92 mg/dL), and a decrease in hemoglobin levels (reaching a minimum of 100 g/L). Concurrently, the mutational load of ccf *BRAF^V600E^* reverted to a positive (0.15%).

Consequently, the treatment regimen was changed to vemurafenib monotherapy (10 mg/kg/dose, administered orally twice daily). Two weeks after the initiation of vemurafenib, follow-up blood tests indicated normalization of hemoglobin and CRP levels and resolution of the skull mass. The plasma concentrations of vemurafenib during the first, second, and third weeks were 11.8, 10.4, and 16.2 μg/mL, respectively. After three months of vemurafenib treatment, there was significant improvement in the cranial and skeletal lesions, and the mutational load of circulating cell-free *BRAF^V600E^* reverted to negative. Later, patients had their peripheral blood tested for ccf *BRAF^V600E^* every 6–8 months, with consistently negative results. The patient stopped vemurafenib in May 2023 after one year and nine months of treatment, experiencing no adverse effects. After stopping vemurafenib, patients had physical exams every 3 months in the first year and every 6 months in the second and third years. At the latest follow-up, 26 months post-therapy., the patient continued to exhibit no signs of active disease (**Figure 2B**).

## 4 Discussion

This study reports the successful management of refractory MS-LCH with vemurafenib monotherapy. The patient’s disease progressed despite treatment with prednisone and vincristine, but showed rapid improvement with vemurafenib monotherapy, which was administered without any side effects. Notably, oral administration of vemurafenib lasted for only one year and nine months, yet the patient remained free of active disease for over two years after cessation of the drug. This case represents the first instance of a patient with refractory MS-LCH treated with vemurafenib monotherapy who did not experience relapse for > 2 years after treatment discontinuation at our center.

The combination of vinblastine and prednisone significantly enhances the overall survival rate of patients with LCH. Conversely, LCH refractory to standard vinblastine and steroid regimens is associated with poor survival outcomes. Although high-intensity chemotherapy or hematopoietic stem cell transplantation can be effective in treating some cases of refractory LCH, these approaches often result in increased toxic side effects (4, 12). Characteristic activation of the MAPK pathway, which involves mutations in genes such as *BRAF*, *MAP2K1*, *ARAF, ERBB3*, *NRAS*, and *KRAS*, provides a rationale for the use of MAPK inhibitors in pediatric patients with LCH (7).

Héritier et al. were the first to report that vemurafenib successfully treated high-risk refractory multisystem LCH in infants (13). However, a multicenter prospective study indicated that although vemurafenib is effective and safe for *BRAF^V600E^*-positive LCH, relapse occurs in 80% of cases after discontinuation (9). Similarly, dabrafenib and trametinib have shown promising results as both first-line and salvage therapies; however, they also exhibit high reactivation upon discontinuation (10, 11). In our center, most patients relapse after discontinuing MAPK inhibitor monotherapy, but this patient remained relapse-free for the longest time. This suggests that other factors may influence post-discontinuation recurrence. Future clinical research and data on non-recurrent patients could help identify factors linked to successful discontinuation and aid in selecting patients for MAPK inhibitors monotherapy to minimize chemotherapy side effects and recurrence.

Understanding the mechanisms that underlie sustained remission following the discontinuation of MAPK inhibitors is crucial for optimizing their use in the treatment of LCH. Eder SK et al. (14) identified that serum inflammatory cytokine levels correspond with vemurafenib treatment, and that RAF inhibition leads to a reduction in the expression of cytokines such as IL1B and CXCL8, which are associated with LCH. Furthermore, genotyping has revealed the presence of the *BRAF^V600E^* mutation in various hematopoietic cells, including natural killer cells and granulocytes, which may influence the morbidity of multisystem LCH. While these findings elucidate the therapeutic effects of targeted drugs on LCH, they do not fully explain why most patients experience relapse after treatment cessation, in contrast to a minority, including our patient, who do not. Investigating the blood and bone marrow of non-recurrent patients through single-cell sequencing could provide valuable insights, presenting an intriguing scientific inquiry.

The optimal timing for the initiation of treatment with MAPK inhibitors in LCH remains unclear. Historically, these inhibitors have been predominantly utilized in cases of refractory or relapsed pediatric LCH, primarily due to safety concerns (9, 11, 13). However, recent studies have demonstrated positive outcomes with frontline trametinib and dabrafenib (10). Furthermore, dabrafenib has been shown to rapidly resolve macrophage activation syndrome hemophagocytic lymphohistiocytosis with reduced toxicity compared with second-line chemotherapy in cases of *BRAF^V600E^*-positive LCH (15). In our study, vemurafenib was employed as salvage therapy in patients resistant to prednisone and vincristine, resulting in rapid disease control without adverse effects such as myelosuppression, hepatic or renal impairment, or infections. MAPK inhibitors have been found to be effective and safe in series clinical trials (9–11), MAPK inhibitors might be potential first-line agents for RO+ MS-LCH, particularly in patients exhibiting organ dysfunction and severe inflammatory responses. The ability of MAPK inhibitors to swiftly stabilize clinical conditions and restore organ function may contribute to a reduction in early mortality and the avoidance of intensive cytotoxic therapies.

In conclusion, although a high rate of relapse has been observed in the majority of patients with LCH following the discontinuation of MAPK inhibitors, our patient have remained in continuous remission even after cessation of medication. Nevertheless, prospective clinical trials are necessary to evaluate the characteristics of patients who do not experience relapse after withdrawal of MAPK inhibitors.

## Data Availability

The raw data supporting the conclusions of this article will be made available by the authors, without undue reservation.
